# The Molecular Context of Oxidant Stress Response in Cancer Establishes ALDH1A1 as a Critical Target: What This Means for Acute Myeloid Leukemia

**DOI:** 10.3390/ijms24119372

**Published:** 2023-05-27

**Authors:** Garrett M. Dancik, Lokman Varisli, Spiros A. Vlahopoulos

**Affiliations:** 1 Department of Computer Science, Eastern Connecticut State University, Willimantic, CT 06226, USA; dancikg@easternct.edu; 2 Department of Molecular Biology and Genetics, Science Faculty, Dicle University, Diyarbakir 21280, Turkey; lokmanv@gmail.com; 3 First Department of Pediatrics, National and Kapodistrian University of Athens, Thivon & Levadeias 8, 11527 Athens, Greece

**Keywords:** acute myeloid leukemia, ALDH1A1, 4-hydroxy-2-nonenal, malondialdehyde, oxidant stress

## Abstract

The protein family of aldehyde dehydrogenases (ALDH) encompasses nineteen members. The ALDH1 subfamily consists of enzymes with similar activity, having the capacity to neutralize lipid peroxidation products and to generate retinoic acid; however, only ALDH1A1 emerges as a significant risk factor in acute myeloid leukemia. Not only is the gene *ALDH1A1* on average significantly overexpressed in the poor prognosis group at the RNA level, but its protein product, ALDH1A1 protects acute myeloid leukemia cells from lipid peroxidation byproducts. This capacity to protect cells can be ascribed to the stability of the enzyme under conditions of oxidant stress. The capacity to protect cells is evident both in vitro, as well as in mouse xenografts of those cells, shielding cells effectively from a number of potent antineoplastic agents. However, the role of ALDH1A1 in acute myeloid leukemia has been unclear in the past due to evidence that normal cells often have higher aldehyde dehydrogenase activity than leukemic cells. This being true, *ALDH1A1* RNA expression is significantly associated with poor prognosis. It is hence imperative that ALDH1A1 is methodically targeted, particularly for the acute myeloid leukemia patients of the poor prognosis risk group that overexpress *ALDH1A1* RNA.

## 1. Introduction

Aldehyde dehydrogenases (ALDHs) are evolutionarily conserved proteins that are involved in the regulation of diverse metabolic processes including proliferation and differentiation, via detoxifying aldehydes to protect cells in both eukaryotes and prokaryotes [1]. In eukaryotic cells, there are 19 ALDH members distributed in several subfamilies [2]. The ALDH1 subfamily members that can oxidize larger substrates as retinol (vitamin A) generate the signaling molecule retinoic acid, which activates its nuclear receptors to regulate gene expression [3,4]. The subfamily member ALDH1A1 (https://www.omim.org/entry/100640) has the capacity to detoxify lipid peroxidation byproducts acrolein, 4-hydroxy-2-nonenal (4-HNE), and malondialdehyde, while it is substantially more resistant to inactivation than the mitochondrial enzyme ALDH2 [5]. 4-HNE causes a decrease in the activity of DNA double-strand break repair proteins, thereby exacerbating DNA damage under oxidative conditions in cells [6]. This means that under conditions of increased oxidant stress ALDH1A1 would be a key enzyme for recovery of the activity of both other aldehyde dehydrogenases, as well as other detoxifying enzymes. ALDH1A1 would allow the additional recovery of other cellular protective mechanisms, including DNA repair that are inactivated by byproducts of lipid peroxidation [7,8].

ALDH activity is crucial for stem cell function in the hematopoietic system, and high ALDH1A1 activity has a role in both protecting hematopoietic stem cells (HSC) from various toxic molecules and also for promoting HSC differentiation, with a partial overlap by ALDH3A1 [9]. Hematopoietic cells from ALDH1A1-deficient mice exhibited increased sensitivity to cyclophosphamide in a non-cell-autonomous manner, consistent with its role in cyclophosphamide metabolism in the liver. However, ALDH1A1 deficiency did not affect hematopoiesis, HSC function, or the capacity to reconstitute irradiated recipients in young or old adult mice [10]. The involvement of ALDH1A1 in HSC differentiation can be attributed to the generation of retinoic acid, as retinoic acid can reverse the block of HSC differentiation by ALDH1A1 inhibition [11].

The partial protective role of ALDH1A1 in HSC biology suggests that it may have a similar role in acute myeloid leukemia (AML) which is a bone marrow malignancy of the myeloid line of blood cells; an abnormal clone that develops from either a myeloid progenitor cell or a myeloblast can give rise to AML, characterized by the rapid growth of abnormal cells that build up in the bone marrow and blood and interfere with normal blood cell production. Indeed, a positive correlation has been shown between low/absent ALDH1A1 activity and favorable prognosis in AML, as expected: patients with AML cells lacking ALDH1A1 had a favorable prognosis, and ALDH1A1^−^ cell lines as well as primary leukemia cells were found to be sensitive to treatment with compounds that directly and indirectly generate toxic ALDH substrates including 4-hydroxynonenal and the clinically relevant compounds arsenic trioxide and 4-hydroperoxycyclophosphamide [12].

ALDH1A1 activity appears indispensable for the survival of AML cells, in contrast to normal HSC [13]. Consequently, targeting ALDH1A1 will probably be an important cornerstone in overcoming therapy resistance in AML.

## 2. Basic Functions of ALDH1 in Acute Myeloid Leukemia

Both types of the ALDH1 enzymatic activities, namely the generation of retinoic acid and detoxification of reactive byproducts are vital for stem cell functions for two reasons: on the one hand, even stem cells that reside in hypoxic niches yield energy in part from oxidative metabolism generating reactive oxygen species, and on the other hand retinoic acid receptors have an essential role in tissue development, including embryogenesis and regeneration, and specifically encompassing morphogenesis and clonogenicity [14,15,16,17,18]. Thus both aspects of ALDH1 enzymatic activity are crucial for stem cells [19].

However, the role of ALDH1A1 in AML has been unclear in the past, due to evidence that normal cells have often higher aldehyde dehydrogenase activity than leukemic cells [20]. Importantly nonetheless, high RNA expression of *ALDH1A1* was recently shown to associate with lower overall survival of AML patients [21]. In view of the properties of the protein product of this gene, and considering the overlap in enzymatic activities between ALDHs, it was vital to investigate the connection between increased RNA expression of ALDH enzyme-encoding genes and the mechanisms that sustain AML stem cells [22]. Despite shared properties between ALDHs, ALDH1A1 emerges as one key member of the ALDH protein family with respect to its anticipated role in acute leukemia [23]. In patents and scientific articles through the nineties, ALDH1A1 was sometimes referred to as ALDH-2, while the mitochondrial isoenzyme ALDH2, which has a substrate selectivity for smaller aldehydes such as acetaldehyde, was referred to as ALDH-1 [24].

**Figure 1:**
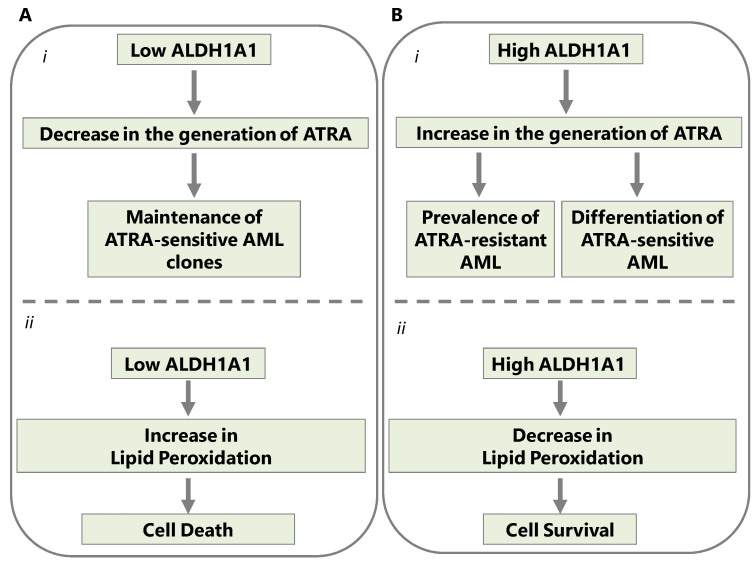
Two main pathways lead to AML progression due to ALDH1A1 overexpression. (**A**) In the AML cells where ALDH1A1 activity is absent or low, (**i**) all-Trans Retinoic Acid (ATRA) does not accumulate and the ATRA-sensitive cell clones persist; nevertheless, (**ii**) lipid peroxidation may kill cells deficient in ALDH1A1 by inducing cellular stress. (**B**) However, when ALDH1A1 is overexpressed in clones of AML cells, (**i**) it generates retinoic acid that will limit the growth of ATRA-sensitive AML clones. Even so, AML clones that have developed resistance to retinoic acid can sustain growth with the added advantage of adaptation to high expression of ALDH1 (ALDH1A1, ALDH1A2, ALDH1A3), and ALDH3B1 (**ii**) ALDH1A1 overexpression in AML cells also allows the cells to survive by detoxifying lipid peroxidation byproducts such as those that are generated by chemotherapeutic drugs.

According to a basic working hypothesis, two are the main paths that lead from ALDH1 overexpression to AML progression: (a) the adaptation of malignant cells to oxidized lipids, and (b) the adaptation of AML cells to increased concentrations of retinoic acid due to alterations in their intracellular signaling apparatus (Figure 1).

## 3. Emerging Role of ALDH1A1 in Cancer Stem Cells: Basic Signaling Pathways

In general, for cancer stem cells ALDH1A1 was proposed as a reporter & marker for self-renewal (clonogenicity), chemoresistance, invasiveness, and metastasis [25,26]. Oxazaphosphorine drugs (such as cyclophosphamide, ifosfamide, and trofosfamide) are broad-spectrum alkylating agents that generate toxic aldehydes; ALDH1A1 contributes significantly to the detoxification of the latter [27]. ALDH1A1 has been proposed to facilitate cancer via the maintenance of cancer stem cell properties, modification of metabolism, and promotion of DNA repair, emphasizing however that ALDH1A1 is not uniformly associated with cancer progression, with several cancer types having a favorable prognosis with higher ALDH1A1 expression [28]. This suggests that the role of ALDH1A1 can become redundant for cancer cells under certain conditions, while it can support essential physiological functions and thereby benefit the host organism.

### 3.1. Regulation of ALDH1A1 in Solid Cancers and Linked Molecular Effects

#### 3.1.1. Non-Small Cell Lung Cancer and Pancreatic Cancer

In non-small cell lung cancer, which is the most common type of neoplasia worldwide, ALDH1A1 is highly expressed after the loss of the aryl hydrocarbon receptor block on carcinogenesis by constitutively active KRAS [29]. In the model for the study of pancreatic intraepithelial neoplasia progression to cancer, deficiency of the regulator ARID1A (AT-rich interaction domain 1A) triggers the expression of ALDH1A1, which enables KRAS-driven carcinogenesis by attenuating cellular senescence (KRAS stands for Kirsten oncogene of rat sarcoma, based on its original discovery) [30]. Hypoxia enables NFκB-driven epithelial–mesenchymal transition, induction of colony and spheroid formation, and ALDH1 activity for pancreatic cancer cells (NFκB stands for nuclear factor κB) [31].

#### 3.1.2. Intrahepatic Cholangiocarcinoma

In the malignant subtype of human intrahepatic cholangiocarcinoma, the loss of ARID1A was an independent prognostic factor for the overall survival of patients (*p* = 0.023); ARID1A and histone deacetylase 1 (HDAC1) were directly recruited to the *ALDH1A1* promoter region in cholangiocarcinoma cells with undetectable *ALDH1A1* expression. ARID1A knockout cells exhibited significantly enhanced migration, invasion, and sphere formation activity and *ALDH1A1* expression [32].

#### 3.1.3. Prostate Cancer

In prostate cancer, inflammatory cues activate I kappa B kinase beta (IKKβ) to phosphorylate ARID1A, leading to its degradation via E3 ubiquitin ligase β-TRCP; ARID1A downregulation, in turn, silences the enhancer of A20 deubiquitinase, a critical negative regulator of NFκB signaling, and thereby unleashes C-X-C motif chemokine receptor 2 (CXCR2) ligand-mediated myeloid-derived suppressor cell chemotaxis that permits cancer progression [33]. In prostate adenocarcinoma, ALDH1A1 shows a statistically significant association with tumor stage (*p* < 0.001), extraprostatic extension (*p* < 0.001), and lymphovascular invasion (*p* = 0.001) [34]. Patterns of mutual exclusivity for ALDH1A1 and ALDH1A3 expression may indicate a certain degree of redundancy between ALDHs in prostate cancer [35]. It is also worth noting that in contrast to cancer cells, stromal cells may lose ALDH1A1 during cancer progression [36].

#### 3.1.4. Ovarian Cancer

In ovarian cancer samples obtained from patients’ ascites, ALDH activity directly correlated to platinum resistance. In the same study in cultured cells, ALDH1A1 knockdown suppressed carboplatin-induced Poly-(ADP-ribose) polymerase (PARP) activity and expression of replication fork associated Fanconi anemia pathway proteins FANCD2 and FANCJ, excision repair protein XRCC1 (X-ray repair cross-complementing 1), replication checkpoint kinase protein 1; while increasing expression of BRCA1 substantially [37]. Ovarian cancer cell lines treated with an ALDH1A1 inhibitor had increased oxidant stress and markers of DNA damage, with diminishing spheroid formation [38].

#### 3.1.5. Breast Cancer, Colorectal Cancer, and Melanoma

Cultured breast cancer cells MDA-2J2 that are resistant to paclitaxel, doxorubicin, staurosporine, and multikinase inhibitor sorafenib, could be sensitized by *ALDH1A1* gene silencing, demonstrating the capacity of ALDH1A1 to mediate resistance to a variety of antineoplastic pharmaceutical agents [39].

Human sarcoma cell line subclones, xenografts, and patient specimens that were selected for resistance to sorafenib had cancer stem cell properties, increased aldehyde dehydrogenase activity, and staining for ALDH1A1 [40]. Sorafenib, a tyrosine kinase inhibitor, is used to treat FMS-related tyrosine kinase-3 internal tandem duplication (FLT3ITD) -positive AML [41,42].

In colorectal cancer stem cells, mutant P53 bound the promoter sequence of the *ALDH1A1* gene, and activated its expression; ALDH1A1 expression was essential to the mutant P53-dependent chemotherapy resistance [43]. Loss of wildtype P53 in cultured cells enriched for cancer stem cell properties including aldehyde dehydrogenase activity and drug efflux, which were reversed by the reintroduction of wildtype P53 [44].

In melanoma cells ALDH1A1, through the retinoic acid pathway, regulated the activation of NFκB and expression of C-X-C Motif Chemokine CXCL8; in co-cultures of melanoma with endothelial cells, the addition of a CXCL8 neutralizing antibody to endothelial cells dampened endothelial angiogenic features, both at the molecular level in terms of gene and protein expression of mediators of the Notch pathway, and at the functional level in terms of proliferation, scratch assay, tube formation and permeability [45].

### 3.2. ALDH1A1 Is Expressed in Hematologic Cancers: Potential for Repression by ARID1A

*ARID1A* is normally also repressed by microRNA223 (mir223), which is transiently activated by NFκB during the late stages of inflammation as a negative feedback gene that terminates inflammation [46,47]. In AML mir223 is essential for leukemia-initiating cells, and at the same time for recovery of the organism from leukemia, for a favorable outcome [48]. Apparently, mir223 becomes redundant once permitting *ALDH1A1* promoter acetylation. Alternatively, ARID1A can be degraded by the proteasome in cells [49]. This could also permit sustained *ALDH1A1* gene expression: AML cells possess significant proteasome activity, which coincides with aggressive disease, and is the main mechanism of NFκB activation because it degrades the inhibitor IκB [50,51,52,53]. In AML, complete remission appears to associate with a rebound in ARID1A expression [54]. Also on the same note, mutations in *ARID1A* are associated with a negative AML course, and in mouse model AML progression [55,56].

In diffuse large B cell lymphoma *ARID1A* mutations do occur [57], which might facilitate *ALDH1A1* expression. Actually, in diffuse large B cell lymphoma, ALDH1A1 enhances phosphorylation of transcription factors STAT3 (signal transducer and activator of transcription 3) and NFκB, augmenting clonogenicity, suppressing caspase activity, and inducing resistance to the chemotherapeutic mixture consisting of cyclophosphamide, doxorubicin, vincristine, and prednisone [58]. ALDH activity alone suffices to select cells that initiate tumors in mice [59].

## 4. AML Stem Cells (LSC) and Their Supporting Network

AML is a heterogeneous disease that arises from immature myeloid progenitor cells in the bone marrow [60]. AML mutations, even those that do not directly control gene expression, co-opt the transcriptional and epigenetic machinery to alter chromatin states, 3D DNA topology, and communication between enhancers and promoters to generate leukemia-specific transcriptional programs; hallmark change is the activation of expression of inflammatory cytokine genes via the transcription factor NFκB [61]. While NFκB is critical for self-renewal of AML, its distinguishing feature in cancer, is that after exposure to cell stress, it becomes activated by intracellular signal pathways that are independent from the cascade that provided the initial carcinogenic NFκB activation [62]. This secondary activation is particularly decisive also in AML, where NFκB drives clonogenicity and drug resistance, regardless of whether the downstream pathway is KMT2A-lysine methyltransferase 2A (MLL)-dependent or homeobox (HOXA)-dependent [63]. Downstream activation of NFκB in patients’ AML cells abolishes response to inhibitors of upstream activating pathways, rendering patients unresponsive [64].

Also in vitro, overexpression of a constitutively active variant of NFκB can rescue stem cell signal-depleted AML cells from apoptosis; this rescue can be attributed to the expression of NFκB downstream target genes such as apoptosis regulator BCL2 [65,66]. Not surprisingly, the BCL2 inhibitor venetoclax appears promising in clinical trials for AML [67,68]. As expected however, mutations in characterized NFκB signaling modulators such as kinase FLT3ITD and K/NRAS (GTPases encoded by respective protoncogenes) predicted inferior response to a venetoclax combination with hypomethylating agents [68]. This result can be explained by an enhanced expression of other targets of NFκB: one such target is oncoprotein mucin1 (MUC1), which supports leukemia cell survival and drug resistance [69,70]. In breast cancer cells, NFκB induced expression of CCAAT/enhancer-binding protein beta (C/EBPβ); the latter formed a complex with MUC1 to activate *ALDH1A1* gene expression [71]. The capacity of NFκB to induce C/EBPβ expression is important for leukemia: ectopic expression of C/EBPβ was shown to activate lymphoid-myeloid transdifferentiation; in particular, when subjected to selective pressure to eliminate lymphoid cells, C/EBPβ-expressing B cells produced granulocyte-macrophage progenitor-like progenitors, which remained self-renewing and cytokine-independent, and continuously produced macrophages and granulocytes [72].

C/EBPβ furthermore can activate antiapoptotic gene expression in synergy with the NFκB subunit P50, which normally functions as a repressor; C/EBPβ namely can disrupt interactions between P50 and histone deacetylases [73]. The diversity in downstream targets demonstrates the critical role of NFκB in the growth and establishment of AML clones. At the same time, it shows the redundancy of signaling cascades in the establishment of AML and suggests that in addition to *BCL2*, other downstream genes such as *ALDH1A1* are also prospective treatment targets.

Leukemia stem cells (leukemia-initiating cells) in AML are considered a key factor in the relapse (recurrence) of AML. In patients, the depletion of leukemia stem cells has a positive prognostic value [74]. It is encouraging that at least under experimental conditions it is possible to target the viability of AML stem cells without affecting normal human stem and progenitor cells substantially [75]. In fact, and despite the redundancy in leukemia stem cell operating mechanisms, interference with NFκB signaling cascades has shown promising clinical results [53,76,77,78]. This is probably due to the presence of constitutively active NFκB in leukemia stem cells, with no comparable constitutive activity in normal hematopoietic progenitor cells [79]. Interference with the expression or activity of downstream NFκB target gene products exposes AML to cell death-inducing cascades; this provides a window of opportunity to disrupt the growth of AML clones [80]. In normal bone marrow, increased inflammatory cytokines induce ALDH1A1 to provide protection from lipid peroxides [81]; in contrast, malignant cells tend to express constitutively high or low levels of ALDH1A1 [82].

**Figure 2:**
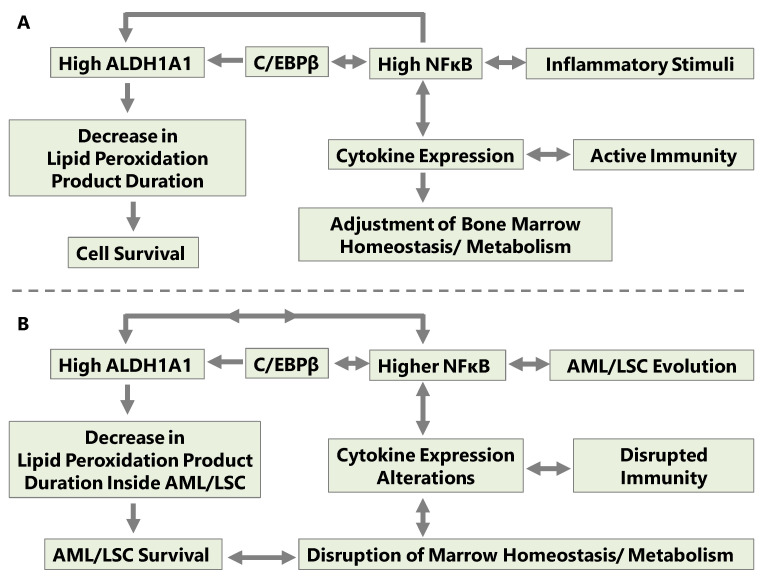
(**A**) Cytokines adjust the metabolism and homeostasis of bone marrow cells. (**B**) AML disrupts marrow function and establishes a niche for malignant cells.

The bone marrow provides a hypoxic niche for hematopoietic stem cells, which can protect both normal progenitors as well as AML cells from endoplasmic reticulum stress through fine-tuning of their metabolic circuitry; this protection functions by a reciprocal exchange of cytokine signals between stromal cells and progenitor cells [83]. During the development of AML, malignant myeloid cells that cause AML initiation occupy the hypoxic bone marrow niche. At a later disease stage, loss of endothelial cells, osteoblasts, HSCs, and the suppression of normal hematopoiesis culminate in niche collapse [84]. AML causes senescence of stromal cells in the bone marrow microenvironment, and senescent mesenchymal cells increase the permissiveness of the bone marrow microenvironment for AML [85,86] (Figure 2).

Internal tandem duplication (ITD) of the *FLT3* gene occurs in 30% of AMLs and confers a poor prognosis. Upon binding with the FLT3 ligand secreted by bone marrow stromal cells, FLT3 undergoes dimerization, phosphorylation, and tyrosine kinase domain (TKD) activation. The internal tandem duplication of FLT3 leads to constitutive activation of downstream survival signals for AML and is targeted by the kinase inhibitor sorafenib. Even though sorafenib is effective, the resistance of AML to sorafenib occurs in patients and can be characterized by increased expression of several genes that include *ALDH1A1*, *JAK3* (Janus kinase 3), and *MMP15* (matrix metallopeptidase 15) [87]. This suggests that products of genes such as *ALDH1A1* could be targeted for the treatment of therapy-resistant AML. Exposure of primary human AML cells to isatin analog KS99 that primarily inhibits ALDH1A1, suppresses clonogenicity, and yields a dose-dependent apoptotic response in primary human leukemic stem cells, identified by the markers CD34^+^, CD34^+^CD38^−^, CD34^+^CD38^+^, CD123^+^, or CD34^+^CD123^+^. It also reduces the leukemic burden in preclinical AML mouse models, both subcutaneous and disseminated xenografts, and improves the survival of animals, especially when combined with cytarabine [88].

Consistently, it was shown that inhibiting ALDH1 activity was sufficient to eradicate AML stem cells, while sparing normal progenitors [13]. This was shown on sorted CD34^+^CD38^−^ subpopulations from AML patients and healthy patients; ALDH1 inhibition was not toxic for healthy HSCs which retained, after treatment, their self-renewing and multi-lineage differentiation capacity in immunodeficient mice, xenografted with human leukemic cells.

In general, in primary disease, the majority of AML cells are associated with a decrease in ALDH activity [89], which however is reversed in cells exposed to cytokines; the cells which gain high constitutive ALDH activity, have a higher leukemia-initiating capacity, and increased drug resistance [19,90]. More specifically, these ALDH1A1 overexpressing cells would be expected to have the capacity to cause relapse after chemotherapeutic treatment in AML. In contrast, before treatment, the ALDH1A1 overexpressing cell clones may be undetectable.

It can be concluded that ALDH1A1 inhibition, targeting primarily leukemia stem cells, could be combined with drugs that target non-stem cells, with the aim to eradicate both quiescent and proliferating AML cell fractions. This approach could curtail AML relapse.

## 5. AML Poor Prognosis and Coordination of Cellular Turnover and Oxidant Stress

*TP53* mutation is the single most adverse prognostic factor in AML, followed by an *FLT3ITD* ratio of mutated to normal alleles (allelic ratio, AR) greater than >0.5 [91,92]. As we discussed above, both features have been linked to increased ALDH activity and to the expression of ALDH1A1. Retinoic acid has shown synergy with the unfolded protein response and oxidative stress to induce cell death in FLT3ITD-positive AML [93]. Retinoic acid has shown a capacity to lower the expression of ALDH1A1 under certain conditions [94].

Mutant P53 protein is expected to lead to aberrant coordination between the cellular metabolic generation of oxidant stress, regulation of cell stress responses, and activation of antineoplastic components of the immune system [95,96,97]. Mutant P53 also interacts with NFκB, and in AML a general dysfunction of P53 signaling underlies the biology of chemoresistance and poor prognosis [98].

In general, AML stem cells appear to tolerate oxidant stress, and increased oxidant stress is associates with a poor prognosis [99,100,101]. FLT3ITD itself causes increased oxidant stress, which triggers antioxidant response cascades that are linked to chemoresistance [102].

Nevertheless, the tolerance of AML cells to oxidant stress is not unlimited, as substantial increases in the production of reactive oxygen species trigger ferroptotic, necroptotic, or apoptotic cell death in AML cell lines and refractory/relapsed AML patient samples [103]. Increased oxidant stress exposes AML cells also to T-cell-mediated immunity [104]. AML cells derive part of their protection from oxidant stress from mitochondria transferred from stromal mesenchymal cells [105]. NADPH oxidase-2 generates superoxide, which stimulates bone marrow stromal cells to transfer mitochondria to AML blasts through AML-derived tunneling nanotubes; this protects AML cells from apoptosis, and causes death to the mammalian host organism, as it was shown by mice that were intravenously injected with patient AML blast cells [106]. In turn, NADP^+^ which is generated both by NADPH oxidases and by the leukemia cells’ antioxidant systems is essential for the activity of ALDH enzymes. Specific patterns of dysregulation of oxidant stress and inflammatory-metabolic regulatory networks are associated with worse clinical outcomes in AML; this is also evident by the fact that levels of advanced oxidation protein products, malondialdehyde, and 8-hydroxydeoxyguanosine are also significantly higher in relapsed AML patients [107,108,109].

8-hydroxydeoxyguanosine (or 7, 8-dihydro-8-oxo-2′-deoxyguanosine, abbreviated 8-oxoG) is normally repaired by 8-oxoguanine DNA glycosylase1 (OGG1) during base excision repair; however high oxidant stress inactivates OGG1, turning it to a coactivator for NFκB-driven expression of genes such as CXCL2 [110]. The CXCL2/CXCR2 axis was associated with increased *ALDH1* expression and chemoresistance in colon cancer cells [111].

CXCL2 supports the growth of AML cells in hypoxia and is linked to poor prognosis of AML [112]. The NFκB-OGG1 interaction, manifest with enhanced *CXCL2* expression, was previously shown to secure the survival of human granulosa-like tumor cells [113]. The interaction of inactivated OGG1 with NFκB, however, elicits the expression of several chemokines and cytokines that contribute to inflammation in addition to enabling innate immunity, they have the capacity to modulate RAS/NFκB dependent carcinogenesis, and to transform the microenvironment of malignant tumors [114,115,116,117]. Adding a selective advantage in a tissue microenvironment under oxidative conditions, ALDH1A1 has the capacity to protect malignant cells from increases in oxidant stress [118]. It has been furthermore correlated to radioresistance, and to markers of epithelial-to-mesenchymal transition [119,120]. ALDH1A1 also contributes to the further activation of NFκB and aggressive cancer properties [45,58].

This event was shown to promote malignant progression in research models for several cancers. For example, a recent study demonstrated how ALDH1A1 regulates the immune system to promote tumor development: high ALDH1A1 increases granulocyte-macrophage colony-stimulating factor (GM-CSF) secretion in an NFκB-dependent manner, leading to myeloid-derived suppressor cell expansion and immunosuppression [121]. They demonstrated the effects of ALDH1A1 in generating a microenvironment that promotes the progression of cancer via NFκB. Although this study was performed in the model of breast cancer, it may reflect a general mechanism that applies to AML and should be further investigated.

In AML, OGG1 is not only associated with adverse cytogenetics, and contributes to cytarabine resistance, but it has a particularly adverse effect on disease outcomes in the context of *FLT3ITD* [122,123]. In fact, a mutant OGG1, namely OGG1 S326C that has a lower threshold of inactivation by oxidant stress, was observed more frequently in patients with AML relapse [28.9 vs. 8.9%, odds ratio = 4.10, 95% confidence interval = 1.35–12.70, *p* = 0.01], and these patients exhibited a shorter relapse-free survival [124].

A high expression of ALDH1A1 in AML cells would be a selective advantage: (a) the enzyme is relatively resistant to inactivation from byproducts of lipid peroxidation, (b) it detoxifies a broad range of aldehyde substrates, particularly long-chain [5], (c) facilitates cell survival by enabling crucial functions of DNA repair [125]. Thus ALDH1A1 will enable cells to rebound after exposure to high levels of oxidant stress and to resume crucial enzymatic activities that are inactivated by free radicals.

Increased levels of malondialdehyde, for example, will cause senescence in the tissue microenvironment, while ALDH1A1-overexpressing AML cells survive, switching readily from their inflammatory state to reactivate DNA repair, and remaining capable to cause disease relapse. In AML, a general drop in ALDH1A1 expression in tissue could coincide with the loss of normal progenitor cells; however, AML stem cells overexpressing ALDH1A1 gain an advantage: on the one hand it protects directly from antineoplastic agents, and on the other hand it reactivates DNA repair, by removing toxic aldehydes that inactivate repair enzymes [126]. AML cells that are resistant to therapeutic treatment show therefore a perturbed response to oxidant stress, characterized by alteration of the patterns of coordination between metabolic function, genome maintenance, and immunity. These conditions are expected to provide an advantage to cell clones that overexpress enzymes such as ALDH1A1. This can explain the association between increased *ALDH1A1* RNA expression and poor prognosis in AML [21].

**Figure 3:**
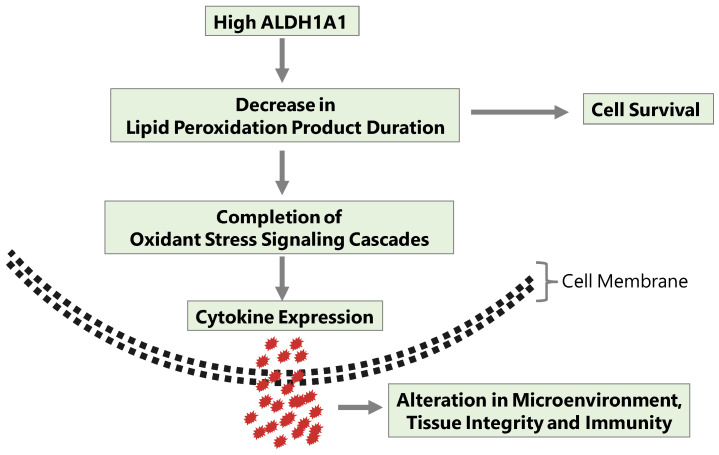
When ALDH1A1 is overexpressed in AML cells, it allows their clones to sustain growth while resisting chemotherapeutic drugs. At the same time, the cells produce a critical level of extracellular mediators that alter tissue integrity and immunity.

To summarize in a concept, initial conditions for AML leukemogenesis would entail a senescent microenvironment with a general deficit in detoxification enzymes, including ALDHs. During progression to advanced AML, increased oxidant stress in the microenvironment, possibly also combined with toxic effects of antineoplastic treatment, select for clones of malignant cells that are resistant to oxidant stress and simultaneously possess the highest leukemia-initiating potential. ALDH1A1 is expected to reach a higher-than-average activity in those leukemia stem cell clones, which thrive under conditions that are toxic to other AML cells. At the same time, oxidant stress elicits changes in the physiology of the affected tissue, which lead to an adverse disease outcome (Figure 3).

## 6. An Agent Development Strategy Can Be Based on the Alteration of NFκB Signaling Cascades in AML

From all of the above, it can be concluded that for patients who belong to the high-risk group, a strategy is needed to neutralize the key factors that provide a selective advantage to leukemia-initiating cell clones. Especially important is the choice of conditions that have the least toxic impact on normal hematopoietic stem cells.

At the heart of this choice is the use of inhibitors for NFκB: either directed on the NFκB complex itself or directed to either upstream and downstream signaling cascades. Direct inhibition of NFκB is achieved by interaction with the glucocorticoid receptor: glucocorticoids indeed have shown activity both against chemoresistant AML cells and also as a clinical treatment for hyperleukocytosis in AML, which is characterized by massive entry of leukemic blasts in peripheral blood [127,128,129].

The use of upstream inhibitors, such as the multikinase inhibitor midostaurin, which in addition to FLT3 inhibits other tyrosine kinases, has proven relatively effective for primary AML [130,131,132]. This has even led to removing *FLT3ITD* from the criteria of patient inclusion to the poor prognosis risk group, in the new, ELN 2022 guidelines: the *FLT3ITD* allelic ratio is no longer considered in the risk classification; consequently, AML with *FLT3ITD* (without adverse-risk genetic lesions) are now categorized in the intermediate-risk group, irrespective of the allelic ratio [133].

The use of downstream inhibitors for the NFκB signaling cascades has proven at least as effective as upstream inhibitors. Specifically, the BCL2 inhibitor venetoclax shows as promising as previously proteasome inhibiting agents [131,134,135]. Proteasome is upstream from NFκB and ALDH1A1, while BCL2 is downstream from NFκB. Primary and adaptive resistance to venetoclax-based combinations was most commonly characterized by the acquisition or enrichment of clones activating signaling pathways such as FLT3 or RAS or biallelically perturbing *TP53* [136]. This led to a combination of venetoclax with FLT3 inhibitors [137]. While venetoclax provided a major advancement in AML treatment, there is still room for improvement.

### 6.1. Interfering with Cell Stress Response Kills AML Cells

Single-agent clinical studies have shown that even the molecule obatoclax, which inhibits both BCL2 as well as its related apoptosis regulator MCL1, is alone not effective in AML, apparently due to the diversity of antiapoptotic mechanisms that AML cells may employ [138]. One such mechanism is autophagy, meaning the degradation of various biomolecules by the lysosomes [139]. FLT3ITD enhances autophagy [140,141]. Autophagy can protect AML cells against a number of both established, as well as last-generation drugs [142]. Elevated autophagic flux, for example, protects AML cells from venetoclax [143]. Autophagy may protect malignant cells that are exposed to cell stress, by degrading cytotoxic molecules and cell-death signal mediators in the lysosome. Common agents that inhibit autophagy include chloroquine and related substances [144]. However, in AML there exists a substantial patient sample heterogeneity in the antileukemic potency of chloroquine, with leukemia-initiating signatures dominating genes expressed in susceptible cells [145]. The susceptibility of leukemia-initiating AML cells to chloroquine can be explained a) by the vital role that lysosome functions have in AML metabolism -especially during leukemogenesis- and b) by a propensity of those lysosomes to be relatively fragile, in comparison to healthy myeloid cells [146].

In this context, ALDH1A1 and HLTF (helicase-like transcription factor) levels seem to be important in the response of cells to hydroxychloroquine (HCQ). In this scenario, HCQ-sensitive cells have high ALDH1A1/low HLTF or low ALDH1A1/low HLTF expression patterns but HCQ-resistant cells have a low ALDH1A1/high HLTF expression pattern [147]. ALDH1A1 increases the influx of lysosomal autophagy inhibitors into the lysosome, whereas HLTF inhibits the oxidant stress-mediated DNA damage caused by lysosomal autophagy inhibitors. Consistently, it must be noted that in AML HLTF has been linked to a favorable prognosis [148].

On the same note, oxidative stress and the ensuing lipid peroxidation have critical effects on various cellular mechanisms and an inhibitory role in autophagy by causing lysosomal dysfunction [149]. In this context, the ALDH1A1 level seems to be critical for recovering autophagy since it detoxifies products of lipid peroxidation. Mechanistically, higher ALDH1A activity leads to enhanced detoxification and consequently further activated autophagy. As a result, increased autophagy will result in a more aggressive and chemotherapy-resistant disease phenotype, as in ALDH1A1-high AML cell populations, which convey a poor prognosis.

A recently identified mediator for ALDH1A1-induced resistance of lung adenocarcinoma cells to tyrosine kinase inhibitors was GPX4 (glutathione peroxidase 4) [118]. This is relevant for AML because GPX4 was also found to protect AML cells from ferroptosis [150]. Glioblastoma cells overexpressing ALDH1A3 could undergo ferroptotic death when exposed to GPX4 inhibitor RSL3 [151].

The selective advantages offered to AML cells by ALDH1A1 under conditions that are detrimental to host tissue can be curtailed by targeting their underlying mechanisms, especially those that pertain specifically to malignant cells. Given that the malignant cells thrive under oxidant stress that switches OGG1 to an NFκB-augmenting factor, the inhibition of this circuit by the compound TH5487 would hamper the interaction of AML cells with their microenvironment [152].

### 6.2. Repurposing Existing Molecules as a Prospective Addition to Treatments of AML

Retinoic acid was proposed as an AML treatment that would decrease ALDH1 activity [153]. In fact, in cultured mouse hepatoma cells, retinoic acid caused feedback inhibition of *ALDH1A1* expression, although retinoic acid receptor alpha acted as an activator: its partnering activator of the *ALDH1A1* promoter, namely C/EBPβ, was repressed by retinoic acid [154]. Thus at least in mouse hepatoma cells, the net effect of retinoic acid on the ALDH1A1 gene promoter was inhibition.

A drug with established broad in vitro activity against neoplastic cells is disulfiram; this drug inhibits multiple pathways, which also include ALDHs [155]. A high-content assay established disulfiram as an inhibitor for ALDH1A1 [156]. It has shown the capacity to kill ALDH-high AML cells, and to overcome resistance to the proteasome inhibitor bortezomib and cytarabine [157,158,159,160]. Not surprisingly, disulfiram prolonged the survival of metastatic non-small cell lung cancer patients in a phase IIb trial [161]. In the organism, disulfiram is gradually metabolized to a number of bioactive compounds, a number of which inhibit ALDH2, the formaldehyde and acetaldehyde oxidizing mitochondrial enzyme [24,162]. However, this does not necessarily marginalize disulfiram use for AML, as the RNA expression of *ALDH2* also has a significant association with unfavorable patient risk, when expression data from over 1000 patients of six leading AML studies were analyzed [21]; thus, both enzymes can be studied as potential targets for AML treatment.

In vivo, the half-life of disulfiram is highly variable and is at best expected to range between 7 and 8 h [163,164,165,166,167]. One solution to this problem may be provided by nanotechnology products, such as encapsulating liposomes [168]. Other agents can be used for encapsulation, which have been used successfully in the past [169] and may gain utility for natural substances in the future [170]. This should permit targeting both ALDH1A1 and ALDH2 for inhibition, thereby increasing the probability of a successful intervention.

## 7. Conclusions

Taken together, a number of mechanisms allow AML cells to survive antineoplastic treatment. In the physiological context, ALDH1A1 is expressed during recovery from inflammation as a mechanism to protect recruited progenitor cells from oxidant stress. The recruited progenitor cells subsequently activate tissue regeneration cascades. The same mechanism that protects essential progenitor cells from oxidant stress, functions in AML to protect leukemia stem cells from byproducts of treatment-induced oxidant stress.

In AML, certain cell subpopulations, and especially leukemia-initiating cells, may adapt to a number of drugs by expressing appropriate proteins that confer drug resistance. In particular, ALDH1A1 enables cancer progression by facilitating drug resistance and fostering resilience of tumor-initiating cells; this applies to cancers driven by RAS/NFκB, FLT3ITD, and mutant P53. On the one hand, this suggests using more than one agent to treat AML, and on the other hand, it suggests monitoring critical mediators of AML treatment resistance, to treat patients accordingly.

The main reason that the ALDH1 role in drug resistance of AML has not been addressed in the clinic, is the prevalence of AML cells with low ALDH1 activity, which made it difficult to understand the dynamic nature of ALDH impact during disease progression. Chemotherapy elicits cellular stress and eliminates sensitive AML clones while selecting clones of therapy-resistant cells. Resistant AML cells give rise to recurrent disease. Disease relapse is thereby driven by cell clones that operate metabolic pathways which allow them to adapt to cellular stress. To kill resistant AML clones, therapeutic intervention needs to interfere with mechanisms of cell stress resistance: one such mechanism is the expression and activity of ALDH1A1.

An improved method of AML treatment thus needs on the one hand to include substances that elicit cellular stress in leukemia cells, such as conventional chemotherapeutic agents, and on the other hand to incorporate substances that inhibit the metabolic adaptation of AML cell clones to cellular stress, as we outline here.

## Data Availability

No new data were created.
